# Brain white-matter changes associated with symptomatic acute COVID-19 infection in the neonatal period

**DOI:** 10.1016/j.idcr.2023.e01796

**Published:** 2023-05-08

**Authors:** Daniel Cromb, Tom Finck, Megan Quirke, Paul Cawley, Amy Moran, Olutoyin Banjoko, Mary A Rutherford, Tomoki Arichi

**Affiliations:** a Centre for the Developing Brain, Department of Perinatal Imaging and Health, School of Biomedical Engineering and Imaging Sciences, Faculty of Life Sciences and Medicine, King’s College London, London, UK; b Department of Neonatology, Evelina London Children’s Hospital, London, UK; c Department of Diagnostic and Interventional Neuroradiology, Klinikum rechts der Isar der Technischen Universität München, Munich, Germany; d MRC Centre for Neurodevelopmental Disorders, King’s College London, London, UK; e Department of Neonatology, Queen Elizabeth Hospital Woolwich, Lewisham and Greenwich NHS Trust, London, UK; f Paediatric Neurosciences, Evelina London Children’s Hospital, London, UK

**Keywords:** COVID, Mri, Magnetic resonance imaging, NEONATOLOGY, Neonatal intensive care, Neonatal neurology

## Abstract

We report an important case of periventricular white matter damage in a 1-month-old infant, demonstrated on high quality structural (T2) and diffusion weighted magnetic resonance imaging. The infant was born at term following an uneventful pregnancy and discharged home shortly after, but was brought to the paediatric emergency department five days after birth with seizures and respiratory distress, testing positive for COVID-19 infection on PCR. These images highlight the need to consider brain MRI in all infants with symptomatic SARS-Cov-2 infection, and show how this infection can lead to extensive white matter damage in the context of multisystem inflammation

We report a male infant born at 38^+4^ weeks, weighing 2.57 kg. His mother was well throughout pregnancy, with no suspicion of antenatal infection, but was diagnosed with SARS-CoV-2 on lateral flow testing shortly after delivery. Both were discharged home within 48 h of the birth. On day five, the infant was admitted via the emergency department with left-sided limb jerking, increased respiratory effort and supplemental oxygen requirement. Multiple EEG-confirmed generalised tonic-clonic seizures were observed over the next 24 h, which terminated following escalating treatment with lorazepam, phenobarbital and, finally, levetiracetam. Empirical broad spectrum antibiotics and acyclovir were started. Serum CRP was < 5 mg/l. Cranial CT and ultrasound scans were normal.

Admission nasopharyngeal swab detected SARS-CoV-2 RNA. Blood, urine and cerebrospinal fluid microscopy and cultures were negative for infection, as was viral serology for Herpes 1 and 2, Varicella Zoster and Enterovirus. Brain MRI at 33 days old revealed extensive white matter injury, presumed secondary to acute SARS-CoV-2 infection, in an otherwise developmentally appropriate brain. He is currently developing well with no neurological concerns.

There are limited reports of neonatal SARS-Cov-2 associated brain injuries on MRI, describing multifocal thalamic T2-hyperintensities in a two-month old infant [1], and restricted diffusion in periventricular and deep white matter in a six-day old infant [2]. Another report describes “seizure-like activity” on day 5 after birth with subcortical and periventricular white matter injury [3].

Neonatologists should have a low threshold for considering brain MRI in infants with symptomatic SARS-CoV2 and associated neurological signs. Extensive white matter injury, suggestive of venous infarctions due to endothelial damage in the context of multisystem inflammation, may be a rare sequelae in an illness that otherwise appears to be well tolerated in the majority of infants (Image 1).

**Image 1:**
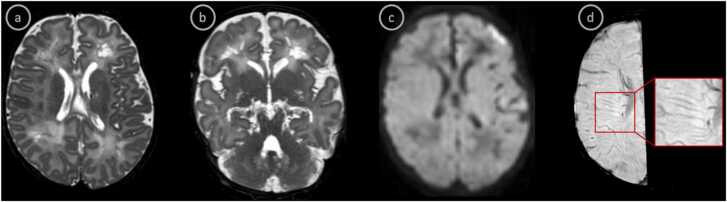
MRI-findings 28 days after symptom onset (aged 43^+1^ postmenstrual weeks). Panels (a) (T2 weighted - axial view) and (b) (T2 weighted - paracoronal view) show symmetrical periventricular white matter cystic infarction following the radiating pattern of draining medullary veins, which are seen on the susceptibility-weighted imaging (SWI) in panel (d). Note the lack of haemorrhage (no susceptibility artefacts on SWI - panel d) and lack of acute ischemic changes (no restricted diffusivity on diffusion weighted Imaging - panel c). Similar patterns of white matter injury may be observed in other RNA-viruses, such as Parechovirus and Rotavirus [4].

## Ethical approval

Data collection, analysis, and anonymous publication of the images and case has been approved by the NHS UK research ethics committee (REC): 19/LO/1384.

## Consent

Written informed consent was obtained from the parents of the patient for publication of this case report and accompanying images as part of a NHS UK research ethics committee approved study (19/LO/1384). A copy of the written consent is available for review by the Editor-in-Chief of this journal on request.

## CRediT authorship contribution statement

**Daniel Cromb:** Conceptualization, Data collection, Data curation, Formal analysis, interpretation, manuscript writing. **Tom Finck:** Data curation, Formal analysis, interpretation, manuscript writing. **Megan Quirke:** Data collection, interpretation, manuscript writing. **Paul Cawley:** Data collection, interpretation, manuscript writing. **Amy Moran:** Data collection, Data curation, manuscript writing. **Olutoyin Banjoko:** Data collection, Data curation, manuscript writing. **Mary A Rutherford:** Conceptualization, Data curation, Formal analysis, interpretation, manuscript writing. **Tomoki Arichi:** Conceptualization, Funding, data collection, Data curation, Formal analysis, interpretation, manuscript writing. All authors have approved the final version of the manuscript for submission.

## Declaration of Competing Interest

The authors have no conflicts of interest to disclose.

## References

[1] Wertheimer G.S.O., Brandao M.B., Reis F. , 2022. COVID-19-related acute necrotizing encephalopathywith new spectroscopy features. Revista da Sociedade Brasileira de Medicina Tropicale 2022; 55: e0275–2022. 10.1590/0037-8682-0275-2022 PMC9536799 36197381

[2] Fragoso D.C., Marx C., Dutra B.G., da Silva C.J., da Silva P.M., Martins Maia Junior A.C., Tobara M.C., Silva C., Silva C.A., Dias L., Polycarpo A.C., Richtmann R. (2021). COVID-19 as a cause of acute neonatal encephalitis and cerebral cytotoxic edema. Pediatr Infect Dis J.

[3] Yildiz H., Yarci E., Bozdemir S.E. (2021). COVID-19-associated cerebral white matter injury in a newborn infant with afebrile seizure. Pedia Infect Dis J.

[4] Verboon-Maciolek M.A., Groenendaal F., Hahn C.D., Hellmann J., van Loon A.M., Boivin G., de Vries L.S. (2008). Human parechovirus causes encephalitis with white matter injury in neonates. Ann Neurol.

